# Acute Pericarditis Secondary to COVID-19 Infection

**DOI:** 10.7759/cureus.20709

**Published:** 2021-12-26

**Authors:** Arminder Singh, Lam Nguyen, Stephanie Everest, Pinang Shastri, Rahel H Alemu

**Affiliations:** 1 Internal Medicine, Cape Fear Valley Medical Center, Fayetteville, USA; 2 School of Medicine, Campbell University School of Osteopathic Medicine, Lillington, USA; 3 Cardiology, Cape Fear Valley Medical Center, Fayetteville, USA; 4 Cardiology, Cape Fear Valley Medical Center, Fayetteville , USA

**Keywords:** transthoracic echocardiography (tte), nsaids, covid pericarditis, pericarditis, recurrent pericarditis

## Abstract

The Coronavirus disease 2019 (COVID-19) pandemic is caused by the severe acute respiratory syndrome coronavirus 2 (SARS-CoV-2). Cardiac injuries are among the complications caused by COVID-19. This report presents the case of a 25-year-old patient hospitalized due to Coronavirus infection with the complication of recurrent acute pericarditis. The patient was treated with colchicine and high-dose ibuprofen, and the patient was then discharged in stable condition. This report demonstrates an effective treatment plan for acute pericarditis secondary to COVID-19 infection.

## Introduction

The novel Coronavirus severe acute respiratory syndrome coronavirus 2 (SARS-CoV-2), also known as Coronavirus disease 2019 (COVID-19), originated in December 2019 in Wuhan, China [1]. SARS-CoV-2 is a large-enveloped, non-segmented positive-sense single-stranded RNA virus that is primarily transmitted via aerosolized droplets [2]. It has ever since rapidly spread worldwide, causing morbidity and mortality along its path. In March 2020, the World Health Organization (WHO) declared the COVID-19 outbreak a pandemic. SARS-CoV-2 is a large enveloped non-segmented positive-sense single-stranded RNA virus transmitted mainly through aerosolized droplets. The most common symptoms of COVID-19 include severe cough, fever, fatigue, anorexia, myalgias, anosmia, dysgeusia, or shortness of breath, and patients with SARS-CoV-2 infection may also present with a sore throat, headache, diarrhea, and rhinorrhea. Moreover, up to 30% of patients who test positive for COVID-19 are asymptomatic [3]. Cardiac injuries, including myocardial infarction, heart failure, arrhythmias, pericarditis, myocarditis, and endocarditis, are seen in approximately 10% of patients [4-7]. COVID-19 patients with cardiac complications have an increased mortality rate compared to those without cardiac involvement, especially in-hospital mortality [6]. Pericarditis is also observed in a minority of patients with SARS-CoV-2 infection, and there are also reports showing significant pericardial effusion or even cardiac tamponade in some of these patients [7,8]. In this report, we describe the clinical presentation, medical management, and outcomes of a case of acute pericarditis secondary to COVID-19 infection.

## Case presentation

**Figure 1:**
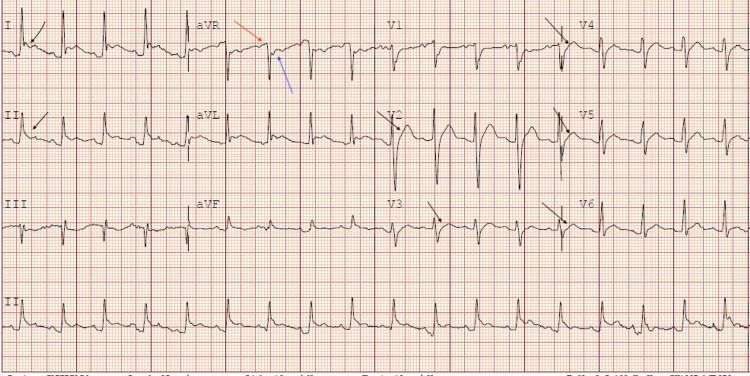
Electrocardiogram on previous admission demonstrating signs of acute pericarditis. Diffuse ST-segment elevation demonstrated by black arrows. Reciprocal PR segment elevation (red arrow) and ST depression (blue arrow) in aVR.

**Figure 2:**
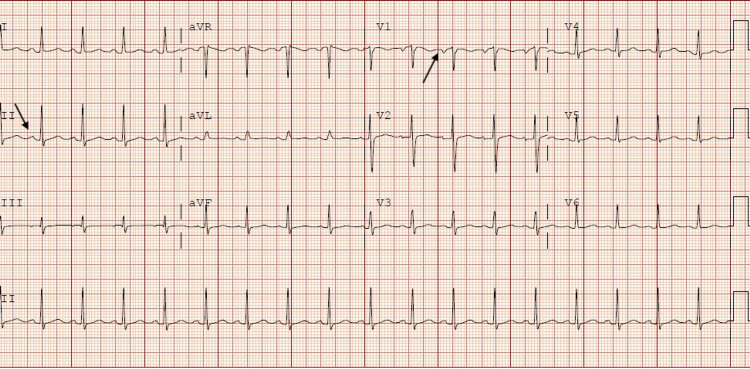
Electrocardiogram on admission demonstrating sinus tachycardia and left atrial enlargement (black arrow indicating P wave enlargement in lead II and lead V1).

**Figure 3:**
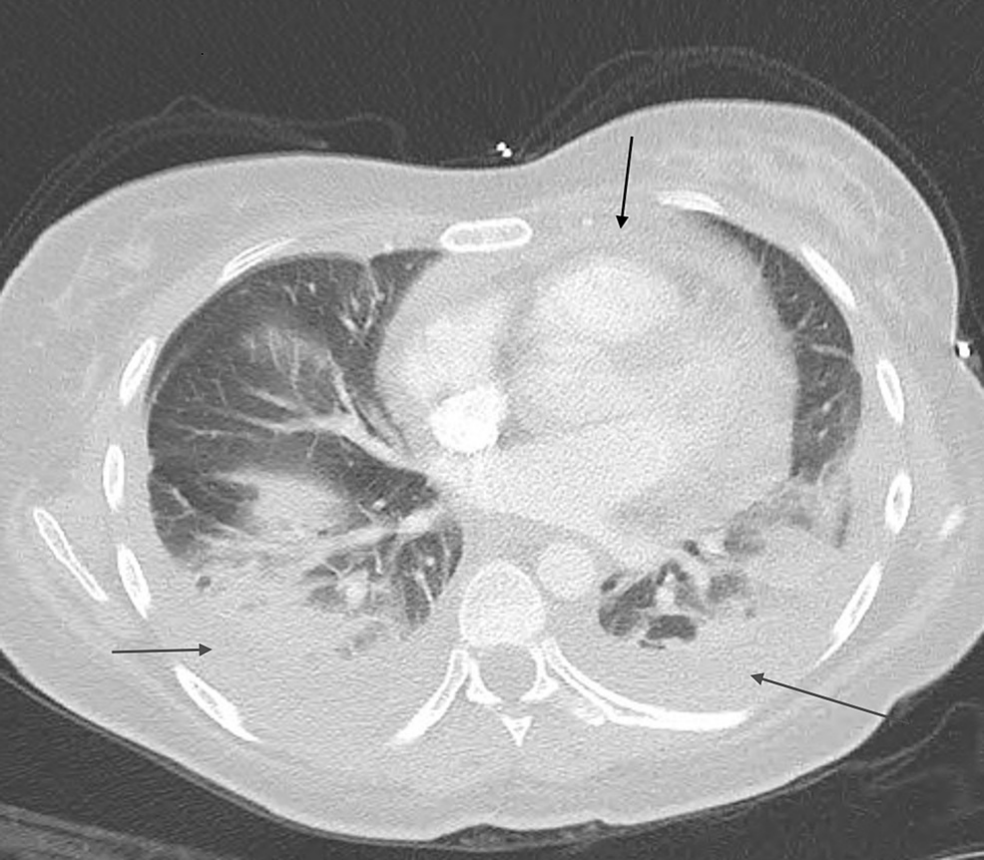
CTA with and without contrast demonstrating no pulmonary embolism, pleural effusions with bilateral basilar atelectasis or consolidation (demonstrated by grey arrows), stable pericardial effusion (demonstrated by black arrow).

A 25-year-old female with a past medical history of anemia, chlamydia, trichomoniasis, and COVID-19 infection presented to the ED with acute respiratory distress, tachycardia, and pleuritic chest pain. The patient had been recently hospitalized one month before presentation for COVID-19 pneumonia with complications of pleural effusion, pericarditis, and superimposed bacterial pneumonia. One month ago, she presented with pleuritic chest pain associated with dyspnea on exertion and a nonproductive cough that had been worsening for five days. She was positive for COVID-19 and was treated with IV Decadron and Remidesivir for COVID-19 pneumonia during the first admission. She was treated with IV antibiotics for superimposed bacterial pneumonia. EKG demonstrated diffuse ST elevations with reciprocal PR segment elevation and ST depression in the aVR lead concerning pericarditis (Figure 1). CRP was more than 190 mg/L, and ESR was 128 mm/HR on the first day of initial admission. The total CK of 153 U/L and the CK-MB of 1.7/mL on the first day of initial admission were within the normal values of the reference range. Troponin on initial admission was negative at less than 0.015 ng/mL. Transthoracic echocardiogram (TTE) obtained on the first admission demonstrated normal systolic function, EF > 55%, mild posterior left ventricular posterior wall thickness, and small pericardial effusion. For the COVID pericarditis, the patient had been started on colchicine 0.6 mg every 12 hours for three months and began high-dose ibuprofen 800 mg every eight hours following improvement in her renal function on the day of discharge. However, the patient never took the high-dose ibuprofen after discharge. Upon hospital arrival, the patient was tachycardic with a heart rate of around 130 beats per minute and tachypneic with respiration of only 30 breaths per minute. Creatinine on admission was 0.62 mg/dL. The COVID test and procalcitonin were both negative. Two sets of troponin on the current admission obtained six hours apart were negative with a value of less than 0.015 ng/mL. An electrocardiogram (ECG) demonstrated sinus tachycardia with left atrial enlargement (Figure 2). A chest radiograph showed small bilateral pleural effusions and bibasilar infiltrates/atelectasis. Computed tomography angiography (CTA) with and without contrast revealed no pulmonary embolism, pleural effusions with bilateral basilar atelectasis or consolidation, or stable pericardial effusion (Figure 3).

**Figure 4:**
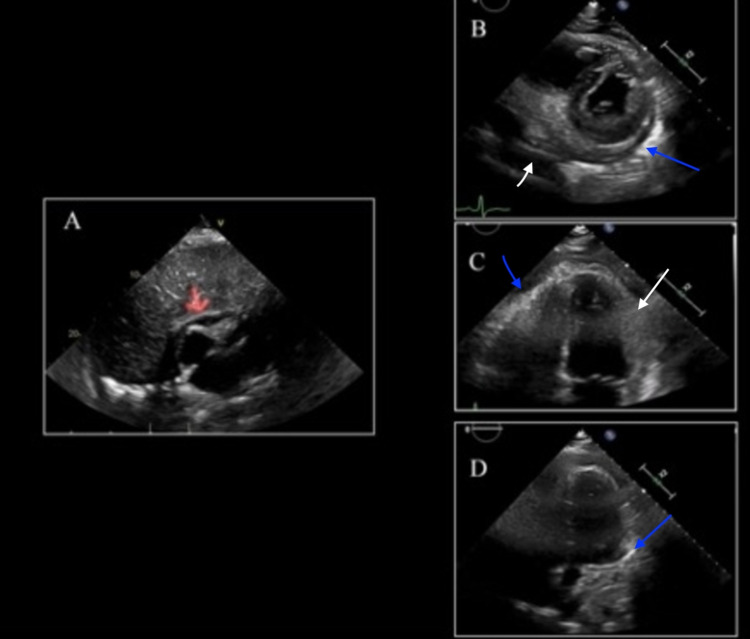
Transthoracic Echocardiogram demonstrating signs of pericarditis. (A) Subcostal view of prior TTE one month ago demonstrating small pericardial effusion with mild echogenicity of the visceral pericardium (demonstrated by red arrow). (B) Short axis view of current TTE demonstrating persistent pericardial effusion (demonstrated by white arrow) with enhanced echogenicity of the visceral pericardium (demonstrated by the blue arrow). (C, D) Four-chamber view of current TTE further demonstrating circumferential thickening (demonstrated by white arrow) and echogenicity of the visceral pericardium (demonstrated by the blue arrow).

The patient was started on antibiotics for suspected bacterial pneumonia, and thoracentesis was not indicated due to the small size of the pleural effusions. The antibiotics for presumed bacterial pneumonia were stopped due to repeat procalcitonin being negative. There was significant thickening of the pericardium seen on the TTE, which appeared organized and was likely to be residual from COVID pericarditis (Figure 4). Also, TTE demonstrated an ejection fraction over 55%, no wall motion abnormalities, no constrictive processes, hyperdynamic systolic function, mildly enlarged right ventricle, mildly reduced right ventricle function, a normal left atrium, and a poorly visible right atrium. Unfortunately, cardiac MRI is not available at the current institution.

For pericarditis, she was started on colchicine 0.6 mg twice day and ibuprofen 800 mg three times daily. Her CRP on admission was 113 mg/L, which improved to 75 mg/L seven days later on discharge after getting started on colchicine and high-dose ibuprofen. Pantoprazole was given with a high dose of ibuprofen for gastric lining protection. The patient was then discharged in stable condition on day 7 of her admission with planned follow-up with cardiology to continue colchicine and high-dose ibuprofen.

## Discussion

Cardiovascular complications such as arrhythmias, hypoxemic cardiomyopathy, pericarditis, myocardial infarction, heart failure, and myocarditis are frequently seen in COVID-19 patients, especially those in the intensive care unit [3-6]. These cardiac injuries could precede or develop during acute respiratory distress syndrome and multiorgan system failure. These cardiac injuries could be the result of direct SARS-CoV-2 effects. The most prominent mediator of this hypothesis is angiotensin-converting enzyme-2 (ACE2) receptors, which are highly expressed in heart and lung tissues [4]. These ACE2 receptors are found to be the functional receptors for the Coronavirus. Another hypothesis for cardiac complications in COVID-19 patients is macrophage-induced inflammation [5]. The SARS-CoV-2 infection leads to invasion of epithelial cells by binding with ACE-2 receptors, localized inflammation, endothelial and macrophage activation, tissue damage, and dysregulated cytokine release [9]. The viral infection activates macrophages, causing them to release massive amounts of cytokines. These cytokines promote the expression of adhesion molecules for inflammatory cell infiltration, endothelial activation, and vascular inflammation. Severe infections are also thought to cause hypoxia and hypoperfusion of the vital organ system, which predisposes patients to cardiovascular insults and thrombotic events [5]. In addition, pharmacologic interventions may be another cause of cardiovascular complications, as antiviral medications are associated with cardiac tissue injuries [4]. 

Pericarditis is defined as inflammation of the pericardium, a double-layered sac surrounding the heart, and pericarditis represents the most common pathological process among pericardial syndromes [10]. Viral infections, including coxsackieviruses, echovirus, adenoviruses, parvovirus B19, HIV, influenza, or herpes viruses, are considered the most common causes of pericarditis [10]. On the other hand, bacterial infections are less likely and frequently cause pericarditis, except for tuberculosis infection in developing countries. Non-infectious causes of pericarditis include malignancy, systemic lupus erythematosus, rheumatoid arthritis, uremia, and myxedema. Clinical manifestations, electrocardiograms, echocardiography, laboratory findings, and advanced imaging can be used for pericarditis diagnosis. For pericarditis management, high dose nonsteroidal anti-inflammatory drugs (NSAIDs) such as Ibuprofen (600 to 800 mg three times a day), Indomethacin (25 to 50 mg three times daily), or Naproxen in addition to colchicine are considered the mainstay treatment regimen. Aspirin (650 to 1000 mg three times a day) can be an alternative to NSAIDs. Colchicine is recommended to be used for 3 to 6 months, and NSAIDs are recommended until symptom relief is achieved. Low to moderate doses of steroids can be used as an alternative in patients with NSAIDs and colochicine contraindications. Furthermore, steroids can also be added to NSAIDs and colchicine as triple therapy for patients with an incomplete response. Third-line treatment and surgical intervention are also available if the options mentioned above fail. 

Our patient presented with typical pericarditis symptoms of chest pain and dyspnea. Her pericarditis was diagnosed based on her clinical presentation, TTE, and CT data. Even though the ECG findings on the current admission were not classic for pericarditis, the clinical presentation and echo findings suggested the pericarditis process. The recurrence of chest pain is likely due to pericarditis due to the patient's returning to the hospital with pleuritic chest pain, TTE findings suggestive of pericarditis, and elevated ESR and CRP inflammatory markers. Sinus tachycardia found on ECG follow-up admission can be a common finding for pericarditis. It is unlikely that her pericardial effusion can be explained by renal dysfunction due to persistent pericardial effusion despite her renal function being at baseline on her last admission and current admission. The patient initially responded well to the single-agent of colchicine, as Ibuprofen was not started. However, the patient was readmitted to hospital care due to a pericarditis recurrence. Fortunately, she was later discharged in stable condition after one week of colchicine and high-dose Ibuprofen treatment. To the best of our knowledge, she has not had any reoccurrences of the pericarditis. The patient's pericarditis recurrence and response to dual therapy of colchicine and Ibuprofen can greatly help understand pericarditis in COVID-19 patients and its treatment. This treatment regimen can also help identify a balanced approach to managing concurrent COVID-19 and its secondary pericarditis. 

## Conclusions

Our patient presented with a less common complication of COVID-19 infection, and she was successfully managed with the recommended therapy for pericarditis. This case report has helped to enhance our understanding of pericarditis in SARS-CoV-2 patients and its treatment. Also, this report can help us establish an effective therapy regimen for both secondary pericarditis and the COVID-19 infection. Further understanding of the pathophysiology and prognosis of this condition will guide clinical treatment decisions in the future. 
